# Structural basis of internal Vangl2 peptide recognition by the Dvl2 PDZ domain

**DOI:** 10.1186/s43556-026-00569-3

**Published:** 2026-09-04

**Authors:** Hanmei Yang, Huaqing Deng, Keru Chen, Ping Zhang, Na Xie, Guobo Shen

**Affiliations:** 1 Department of Biotherapy, Cancer Center and State Key Laboratory of Biotherapy, Medicine, West China Hospital, and West China School of Basic Medical Sciences and Forensic, Sichuan University, Chengdu, 610041 China; 2 Chengdu Integrated TCM & Western Medicine Hospital, Chengdu, 610041 China

**Dear Editor,**

Planar cell polarity (PCP) signaling is an evolutionarily conserved pathway that regulates cellular asymmetry and tissue organization during development and homeostasis. Dysregulation of PCP signaling is associated with developmental defects and human diseases, highlighting its essential biological functions. Core PCP proteins, including Van Gogh-like 2 (Vangl2) and Dishevelled (Dvl), form dynamic molecular complexes through regulated protein–protein interactions to control PCP signal transduction [1]. PDZ domains typically recognize short C-terminal peptide motifs (PDZ-binding motifs, PBMs) through interactions involving the terminal carboxyl group. However, structural studies have demonstrated that certain PDZ domains, including Dvl PDZ domains, can also recognize internal peptide sequences, revealing broader ligand recognition capabilities [2]. Although Dvl PDZ domains are capable of binding internal ligands, the structural basis underlying recognition of an internal Vangl2 sequence remains unclear. Here, we identify an internal Vangl2 peptide (residues 302–311) as a direct binding element for the Dvl2 PDZ domain and define the molecular basis of this interaction through X-ray crystallography, quantitative binding analyses, reciprocal mutagenesis, and cellular validation.

To identify the region of Vangl2 responsible for Dvl2 binding, we performed truncation mapping using pull-down assays. Progressive deletion analysis identified residues 292–340 within the cytoplasmic region of Vangl2 as the minimal region required for interaction with the Dvl2 PDZ domain (Fig. 1a). Further analysis demonstrated that the internal sequence spanning residues 302–311 is critical for recognition. We subsequently determined the crystal structure of the Dvl2 PDZ domain bound to the Vangl2 (302–311) peptide at 1.95 Å resolution (Table S1). The asymmetric unit contains four independent Dvl2 PDZ–Vangl2 peptide complexes with highly similar conformations, and the composite omit electron density map supports the modeled peptide structure. The Vangl2 peptide adopts an extended conformation and occupies the canonical peptide-binding groove of the Dvl2 PDZ domain (Fig. 1b). Unlike classical PDZ ligands that engage through a free C-terminal carboxyl group, Vangl2 is recognized through an internal peptide sequence, expanding the known ligand recognition modes of Dvl PDZ domains. The interface is stabilized by hydrophobic and polar interactions, including an electrostatic interaction between Lys306 of Vangl2 and Asp331 of Dvl2, together with hydrophobic contacts involving Tyr308 (Fig. 1c).

**Fig. 1:**
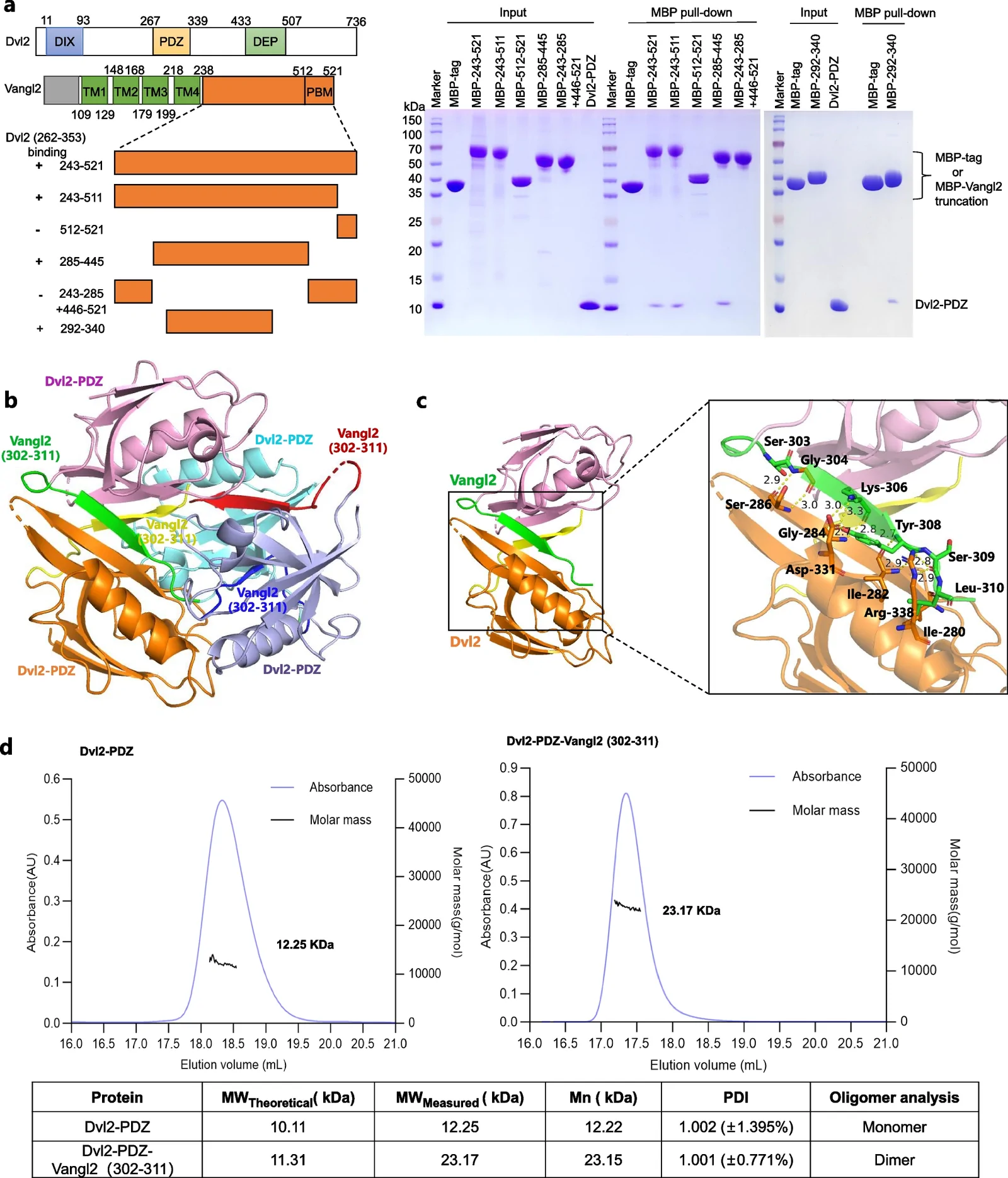
Structural and biochemical characterization of the Vangl2–Dvl2 interaction. **a** Schematic representation of Vangl2 domain organization and truncation constructs used for interaction mapping. Pull-down assays were performed to evaluate the interaction between Dvl2-PDZ and Vangl2 fragments. Residues 292–340 of Vangl2 were identified as the minimal region required for interaction with the Dvl2 PDZ domain. **b** Crystal structure of the Dvl2 PDZ domain bound to the Vangl2 (302–311) peptide determined at 1.95 Å resolution. The Vangl2 peptide adopts an extended conformation and occupies the canonical peptide-binding groove of the Dvl2 PDZ domain. Four independent complexes are present in the asymmetric unit and exhibit highly similar peptide-binding conformations. **c** Close-up view of the Dvl2 PDZ–Vangl2 peptide interaction interface. The key residues involved in the interaction are depicted as stick models, with hydrogen bonds denoted by dashed lines. An electrostatic interaction between K306 of Vangl2 and D331 of Dvl2, together with hydrophobic contacts involving Y308 and surrounding residues, contributes to peptide recognition. **d** SEC–MALS analysis of the isolated Dvl2 PDZ domain and the engineered Dvl2 PDZ–Vangl2 fusion construct. The isolated Dvl2 PDZ domain behaves as a monomer with a measured molecular weight of approximately 12 kDa, whereas the engineered fusion construct exhibits an apparent dimeric species with a measured molecular weight of approximately 23 kDa. This dimeric behavior is consistent with the domain-swapped arrangement observed in the crystal structure and reflects the architecture of the engineered fusion construct

To evaluate the solution behavior of the crystallized construct, we performed SEC–MALS analysis. The isolated Dvl2 PDZ domain behaved as a monomer in solution, whereas the engineered Dvl2 PDZ–Vangl2 fusion construct exhibited an apparent dimeric species (Fig. 1d). This observation is consistent with the domain-swapped arrangement observed in the crystal lattice, where the Vangl2 peptide from one fusion molecule engages the PDZ domain of another molecule. However, this apparent dimerization likely reflects the architecture of the engineered construct and does not establish the oligomeric organization or stoichiometry of the native full-length Vangl2–Dvl2 complex.

To independently validate the interaction interface and exclude artifacts introduced by the fusion strategy, we performed BLI assays using chemically synthesized free Vangl2 peptides and the isolated Dvl2 PDZ domain. The wild-type Vangl2 (302–311) peptide directly bound Dvl2 PDZ with an equilibrium dissociation constant (KD = 172 ± 22 μM, fitting error). Reciprocal mutational analyses demonstrated that mutations of Vangl2 interface residues (F305A, K306D, Y308A, and L310A) markedly impaired binding, whereas mutations within the Dvl2 PDZ domain (D331K, R338A, and I282A/G284A/S286A) also reduced recognition of the wild-type peptide. Furthermore, Co-immunoprecipitation followed by Western blot analysis using full-length proteins showed that structure-guided mutations in either Vangl2 or Dvl2 significantly weakened their interaction in cells, providing independent cellular support for the identified binding interface.

PDZ domains are increasingly recognized as flexible interaction modules that mediate transient protein associations rather than exclusively forming stable complexes [3, 4]. Although the affinity between the isolated Vangl2 peptide and Dvl2 PDZ domain is relatively modest (KD ≈ 172 μM), micromolar-affinity PDZ interactions are frequently observed in signaling pathways. For example, interactions between Dvl PDZ domains and Frizzled-derived peptides have also been reported within the micromolar range, while membrane localization and multivalent interactions can enhance complex formation in cells [4]. Thus, the measured affinity is compatible with a dynamic regulatory interaction within PCP-associated complexes.

Our data further demonstrate that the Vangl2 internal motif, rather than its predicted C-terminal PBM, mediates binding to Dvl2 PDZ, indicating that sequence context and structural complementarity, rather than motif presence alone, determine PDZ ligand specificity. The key interface residues are conserved in Vangl1 and Dvl family members, suggesting evolutionary conservation of this recognition mechanism. Interestingly, several potential phosphorylation sites, including Ser303, Tyr308, and Ser309, have been annotated within the identified Vangl2 internal peptide region in the PhosphoSitePlus database. These modifications may represent an additional regulatory layer influencing peptide properties, Dvl2 PDZ recognition, or Vangl2–Dvl2 complex assembly; however, their functional consequences remain to be determined through future biochemical and cellular studies [5].

In summary, we identify an internal Vangl2 peptide as a direct recognition element for the Dvl2 PDZ domain and define the molecular basis of this interaction through structural, biochemical, and cellular approaches. These findings expand our understanding of PDZ-mediated molecular recognition and demonstrate how internal peptide interactions contribute to the dynamic regulation of PCP signaling complexes. Future structural and functional studies of full-length Vangl–Dvl assemblies will be important for determining how this interaction contributes to PCP regulation in vivo.

## Supplementary Information

Supplementary Material 1.

## Data Availability

The atomic coordinates and structure factors for the Vangl2-Dvl2 complex have been deposited in the Protein Data Bank (PDB) under accession code 35UG. All data generated or analyzed in this study are available from the authors upon reasonable request.
