# Supplementary material for: Structural basis of internal Vangl2 peptide recognition by the Dvl2 PDZ domain
Source: Mol Biomed. 2026 Sep 4;7:160. doi: 10.1186/s43556-026-00569-3 (PMC13542021; doi:10.1186/s43556-026-00569-3)
Supplement: Supplementary file 1 [file 43556_2026_569_MOESM1_ESM.docx]

**Supplemental information:**

**Methods**

**Expression and purification of truncated Vangl2**
We subcloned the codon‑optimized cDNA encoding the human Vangl2 tail region (residues 292–521) into the pFastBac1 vector, which added an N‑terminal MBP tag. The Bac‑to‑Bac system was employed to generate recombinant baculoviruses, which were subsequently amplified in Sf9 insect cells. These cells were infected with P3 virus stock and maintained at 27 °C for 72 h. After harvest, the cells were disrupted by ultrasonication in lysis buffer (20 mM Tris‑HCl, pH 8.0, 150 mM NaCl, 1 mM PMSF, 5 % glycerol), and the cleared supernatant was incubated with Amylose Resin (NEB) at 4 °C for 1 h. After washing, the bound fraction was eluted with buffer containing 15 mM maltose and then subjected to size‑exclusion chromatography on a Superdex 200 Increase column pre‑equilibrated with 20 mM Tris‑HCl (pH 8.0) and 150 mM NaCl.

**Recombinant production and purification of Dvl2‑PDZ and its mutants**
For the wild‑type and mutant Dvl2‑PDZ (residues 262–353), the codon‑optimized cDNA was inserted into pGEX‑6p‑1, which introduced a GST tag at the N‑terminus and a 6×His tag at the C‑terminus. The resulting plasmid was transformed into BL21(DE3) *E. coli*, and protein production was induced with 0.5 mM IPTG at 16 °C for 18 h. Bacterial pellets were lysed by sonication, and the soluble lysate was mixed with Glutathione Sepharose 4B beads (Cytiva) for 1 h at 4 °C. After thorough washing, the GST moiety was removed on‑column using 3C protease at 4 °C overnight. The cleaved target protein was eluted and further polished via size‑exclusion chromatography on a Superdex 200 Increase column equilibrated with 20 mM Tris‑HCl (pH 8.0) and 200 mM NaCl. Mutants were constructed by site‑directed mutagenesis using the wild‑type plasmid as template and purified using the same protocol.

**Protein pull-down and Co-immunoprecipitation (Co-IP) assays**

Each of the six truncated MBP-Vangl2 proteins (20 μg) and MBP alone (20 μg) was incubated separately with Dvl2-PDZ (16 μg) at 4 °C for 1 h, followed by the addition of 20 μL Amylose Resin (NEB) and incubation for an additional 1 h at 4 °C. The beads were washed three times with wash buffer (20 mM Tris-HCl, pH 8.0, 150 mM NaCl). Bound proteins were eluted with 40 μL elution buffer (20 mM Tris-HCl, pH 8.0, 150 mM NaCl, 15 mM maltose) and resolved by 12% SDS-PAGE.

HEK293 cells expressing full-length FLAG-Vangl2 (1–521) or C-terminally truncated FLAG-Vangl2 (1–512) were lysed and incubated with purified GST-Dvl2 PDZ protein, followed by Glutathione Sepharose 4B beads (17075601, Cytiva). Following thorough washing, the captured proteins were released and then subjected to SDS‑PAGE and immunoblotting using anti‑GST‑HRP (#5475, CST) and anti-FLAG-HRP (M185-7, MBL) antibodies.

HEK293 cells were co-transfected with full-length FLAG-VANGL2 and DVL2-HA constructs (wild-type or mutants). At 48 h post‑transfection, the cells were collected, lysed, and subjected to immunoprecipitation with anti‑FLAG G1 affinity gel (L00432, GenScript) or anti-HA affinity gel (L00777, GenScript). The beads were washed extensively, and the bound fraction was eluted for analysis by SDS‑PAGE and immunoblotting with anti-FLAG-HRP (M185-7, MBL) and anti-HA-HRP (1008-3, Huabio) antibodies.

**Expression and purification of Dvl2-PDZ-Vangl2 (302–311) fusion construct**

The codon-optimized cDNAs encoding human Dvl2 (residues 262–353) and Vangl2 (residues 302–311) were inserted into a modified pET28a vector carrying an N‑terminal 6×His‑SUMO tag. After transformation into E. coli BL21(DE3), expression was triggered by adding 0.5 mM IPTG and incubating at 16 °C for 18 h. The fusion protein was captured using Ni-NTA affinity beads, and the 6×His-SUMO tag was removed on-column using TEV protease at 4 °C overnight. Size‑exclusion chromatography (Superdex 200 Increase) was employed for further purification of the eluted untagged protein, with the column pre‑equilibrated in 20 mM Tris‑HCl (pH 8.0) and 200 mM NaCl.

**Protein crystallization, X-ray diffraction data collection and processing**

The purified Dvl2-PDZ-Vangl2 (302–311) protein was concentrated to 12 mg/mL for crystallization screening using the sitting-drop vapor-diffusion method at 20 °C. Crystals were obtained under optimized conditions containing 0.1 M HEPES (pH 7.5) and 24% (w/v) PEG 10000. Prior to data collection, crystals were cryoprotected by immersion in 25 % ethylene glycol and rapidly frozen in liquid nitrogen. X-ray diffraction data were collected at the BL02U1 beamline of the Shanghai Synchrotron Radiation Facility with a 1° oscillation step over a total rotation range of 360°. The structure was determined by molecular replacement using the Dvl2 PDZ domain structure (PDB ID: 3CBZ) as the search model, with the bound peptide removed, implemented in PHASER. Model building and refinement were performed iteratively using COOT and PHENIX. Structural figures were generated using PyMOL. The final structure was refined to 1.95 Å resolution and deposited in the PDB under accession code 35UG. Statistics related to data collection and refinement can be found in Supplementary Table S1.

**Bio-Layer Interferometry (BLI)**

BLI assays were performed on an Octet RED96 system (ForteBio) at 25 °C. Wild-type Dvl2-PDZ and mutants (60–100 nM) were immobilized onto Ni-NTA biosensors (ForteBio) for 60 s in binding buffer (20 mM Tris-HCl, pH 8.0, 200 mM NaCl, 0.05% Tween-20). Synthetic Vangl2 (302–311) peptides (wild-type or mutants) were serially diluted in binding buffer (50–1600 μM) and used as analytes. Association and dissociation were monitored, and binding curves were globally fitted to a 1:1 binding model using ForteBio Data Analysis HT 12 software. All measurements were performed once; errors represent the point-to-point average deviations between experimental data and fitted curves.

**SEC‑MALS analysis**

SEC‑MALS experiments were carried out on an AKTA pure system coupled with a Wyatt Dawn Heleos II MALS detector. Protein samples (100 μg) were loaded onto a Superdex 200 Increase column equilibrated with PBS and eluted at 0.5 mL/min at 25 °C. Data were acquired and analyzed using Astra 8 software (Wyatt Technology) to determine absolute molecular mass and oligomeric state.

**All data generated or analyzed in this study are available from the authors upon reasonable request.**

**Table S1** The x-ray crystal lographic data collection and refinement statistics

| **Dataset** | **Vangl2-Dvl2** |
| --- | --- |
| **Data collection** | |
| Beamline | 02U1 |
| Wavelength | 0.979183 |
| Resolution (Å) | 36.61-1.95 (2.02-1.95) |
| Space group | P 1 2_1_ 1 |
| a, b, c (Å) | 48.33，52.32，77.52 |
| α, β, γ (°) | 90.00，104.21，90.00 |
| Total reflections | 214098 (11613) |
| Unique reflections | 33128 (2417) |
| Multiplicity | 6.5(4.8) |
| Completeness (%) | 98.1% (96.8%) |
| Mean I/sigma (I) | 10.19 (at 1.95 Å) |
| Refinement program | PHENIX1.13_2998 |
| Wilson B-factor ( Å^2^) | 20.9 |
| Anisotropy | 0.234 |
| R-merge | 0.055 (0.304) |
| R-meas | 0.060 (0.341) |
| R-pim | 0.023 (0.151) |
| CC1/2 | 0.999 (0.938) |
| **Refinement** | |
| Reflections used in refinement | 27047 (2646) |
| R_work_ | 0.185 |
| R_free_ | 0.228 |
| Number of non-hydrogen atoms | 3253 |
| Macromolecules | 2995 |
| Solvent | 258 |
| Protein residues | 406 |
| RMS (bonds) | 0.0026 |
| RMS (angles) | 0.57 |
| Ramachandran favored (%) | 97.44 |
| Ramachandran allowed (%) | 2.56 |
| Ramachandran outliers (%) | 0 |
| Rotamer outliers (%) | 0.610 |
| Clash score | 0.83 |
| Average B-factor ( Å^2^) | 32.0 |
| Macromolecules | 0.87 |
